# The role of the gut microbiota on the metabolic status of obese children

**DOI:** 10.1186/s12934-021-01548-9

**Published:** 2021-02-27

**Authors:** Xin Yuan, Ruimin Chen, Kenneth L. McCormick, Ying Zhang, Xiangquan Lin, Xiaohong Yang

**Affiliations:** 1 Department of Endocrinology, Fuzhou Children’s Hospital of Fujian Medical University, NO. 145, 817 Middle Road, Fuzhou, 350005 China; 2 Division of Pediatric Endocrinology and Diabetes, University of Alabama at Birmingham, Birmingham, AL 35233 USA

**Keywords:** Metabolically healthy obese, Children, 16s rRNA, Gut microbiota

## Abstract

**Background:**

The term “metabolically healthy obese (MHO)” denotes a hale and salutary status, yet this connotation has not been validated in children, and may, in fact, be a misnomer. As pertains to obesity, the gut microbiota has garnered attention as conceivably a nosogenic or, on the other hand, protective participator.

**Objective:**

This study explored the characteristics of the fecal microbiota of obese Chinese children and adolescents of disparate metabolic statuses, and the associations between their gut microbiota and circulating proinflammatory factors, such as IL-6, TNF-α, lipopolysaccharide-binding protein (LBP), and a cytokine up-regulator and mediator, leptin.

**Results:**

Based on weight and metabolic status, the 86 Chinese children (ages 5–15 years) were divided into three groups: metabolically healthy obese (MHO, n = 42), metabolic unhealthy obese (MUO, n = 23), and healthy normal weight controls (Con, n = 21). In the MUO subjects, the phylum *Tenericutes*, as well as the alpha and beta diversity, were significantly reduced compared with the controls. Furthermore, Phylum *Synergistetes* and genus *Bacteroides* were more prevalent in the MHO population compared with controls. For the MHO group, Spearman’s correlation analysis revealed that serum IL-6 positively correlated with genus *Paraprevotella*, LBP was positively correlated with genus *Roseburia* and *Faecalibacterium*, and negatively correlated with genus *Lactobacillus,* and leptin correlated positively with genus *Phascolarctobacterium* and negatively with genus *Dialister* (all p < 0.05).

**Conclusion:**

Although there are distinct differences in the characteristic gut microbiota of the MUO population versus MHO, dysbiosis of gut microsystem is already extant in the MHO cohort. The abundance of some metabolism-related bacteria associates with the degree of circulating inflammatory compounds, suggesting that dysbiosis of gut microbiota, present in the MHO children, conceivably serves as a compensatory or remedial response to a surfeit of nutrients.

**Supplementary Information:**

The online version contains supplementary material available at 10.1186/s12934-021-01548-9.

## Introduction

The global epidemic of childhood obesity, and the accompanying rise in the prevalence of endocrine, metabolic, and cardiovascular comorbidities, is perhaps the most impactful and ubiquitous public health disorder of the modern world [1]. In the context of this pandemic, a distinct group of youth with obesity who are devoid of metabolic disturbances—so-called “metabolically healthy obese” (MHO)—have been identified. Obesity notwithstanding, by definition MHO children retain a favorable metabolic profile, with preserved insulin sensitivity along with normal blood pressure, glucose homeostasis, lipids, and liver enzymes. Moreover, their hormonal, inflammation, and immune profiles are seemingly impervious to obesity [2]. First described in obese adults, the MHO phenotype has also been extensively studied in young people with obesity [2]. Arguably, MHO may be a transitional stage to the far more common, more high-risk, conventional cardio-metabolic obese phenotype. Regardless of the aforesaid normal biochemical characteristics of MHO, the risk for cardiovascular disease persists since the MHO phenotype may be unstable, thereby transitory [3, 4].

Among the non-genetic factors associated with obesity, the gut microbiota has garnered attention as an obesity regulator given the robust correlations in animal studies between gut microbiota and body weight. Obese individuals, whether adults or children, have increased abundance in *Firmicutes* in concert with decreased in *Bacteroidetes* [5, 6]. The distinctive gut microbiota prevalent in obese subjects is recognized as promoting an unhealthy metabolic obese (MUO) phenotype with attendant comorbidities, such as increased endotoxemia, intestinal and systemic inflammation, as well as insulin resistance. An altered gut microbiota has been implicated in obesity and type 2 diabetes mellitus (T2DM) insofar as a decrement in certain species and gene richness have been linked to adiposity, dyslipidemia, and insulin resistance [7]. Hence, the clinical repercussions aside, it is plausible that differences in the gut microbiota could dictate whether an obese child is metabolically fit (MHO) or not (MUO) [8, 9].

Obesity and related metabolic disorders are associated with gut microbiota dysbiosis, disrupted intestinal barrier and chronic inflammation [10]. For instance, obese Mexican children and adolescents had increased levels of leptin and C-reactive protein, which were associated with changes in the gut microbiota [11]. However, the association between gut microbiota and proinflammatory cytokines, such as IL-6, TNF-α and lipopolysaccharide-binding protein (LBP), has not been fully investigated in children of varying metabolic statues. Firstly, this study examined the metabolic heterogeneity of obese children as it relates to the composition of the gut microbiota. And, as a secondary end point, identify metabolic-specific bacteria which associate with serum inflammatory factors incriminated in obesity comorbidities.

## Results

### Study participants

Based on weight status, the metabolically stable cohort subjects (n = 63) were subdivided as MHO (n = 42) or Con (n = 21).

The age of the 86 participates ranged from 5.5 to 14.3 years, with a mean of 9.76 ± 1.93 years. There were 65 obese children, of whom 23 were MUO and 42 were MHO. The BMI of other 21 children were normal. Age, weight, BMI, BMI-Z, WHtR, SBP, TG and LDL-c in the MUO group were significantly higher than the Con and MHO children, and HDL-c in the in the MUO group were significantly lower than the Con and MHO children (all p < 0.05, Table 1).

**Table 1:** Anthropometric profiles and laboratory measurements

	MUO (n = 23)	Metabolic healthy subjects		
		Total (n = 63)	MHO (n = 42)	Con (n = 21)
Age (year)	10.96 ± 1.69	9.32 ± 1.84*	9.47 ± 1.68*	9.02 ± 2.14
Male (%)	65.2	50.8	54.8	42.9
Weight (kg)	61.4 ± 11.5	43.0 ± 14.6*	49.6 ± 12.4*	29.9 ± 8.5^#^
BMI (kg/m^2^)	27.02 ± 2.75	21.80 ± 4.91*	24.65 ± 3.14*	16.11 ± 1.91^#^
BMI-Z	2.81 ± 0.61	1.77 ± 1.53*	2.74 ± 0.60	− 0.16 ± 0.79^#^
WHR	0.89 ± 0.05	0.86 ± 0.06	0.88 ± 0.05	0.84 ± 0.06^#^
WHtR	0.55 ± 0.04	0.50 ± 0.06*	0.53 ± 0.04	0.43 ± 0.03^#^
SBP (mmHg)	116.45 ± 8.77	101.52 ± 8.36*	105.51 ± 6.96*	94.48 ± 5.51^#^
DBP (mmHg)	65.09 ± 5.72	62.57 ± 5.79	63.81 ± 6.45	60.38 ± 3.56#
FPG (mmol/L)	5.09 ± 0.67	4.87 ± 0.39	4.82 ± 0.38*	4.97 ± 0.40
TC (mmol/L)	4.54 ± 0.90	4.30 ± 0.62	4.39 ± 0.57	4.14 ± 0.69
TG (mmol/L)	1.62 ± 0.99	0.86 ± 0.30*	0.93 ± 0.33*	0.72 ± 0.19^#^
LDL-c (mmol/L)	2.65 ± 0.66	2.31 ± 0.53*	2.45 ± 0.48	2.03 ± 0.54#
HDL-c (mmol/L)	1.24 ± 0.24	1.58 ± 0.30*	1.51 ± 0.30*	1.71 ± 0.26^#^
Leptin (μg/mL)	2.70 ± 1.48	2.23 ± 1.83	3.10 ± 1.65	0.51 ± 0.35*^#^
TNF-α (pg/mL)	47.50 ± 25.63	48.48 ± 18.77	53.43 ± 17.88	38.59 ± 16.81^#^
IL-6 (μg/mL)	1.76 ± 0.86	1.65 ± 0.93	1.86 ± 1.04	1.23 ± 0.42*^#^
LBP (μg/mL)	34.8 (29.55, 41.20)	33.66 (27.01, 38.95)	33.28 (27.75, 41.22)	27.18 (22.02, 36.61)*^#^

*MUO* metabolic unhealthy obese, *MHO* metabolically healthy obese, *Con* controls, *BMI* body mass index, *BMI-Z* BMI standard deviation Z score, *WHR* waist-to-hip ratios, *TC* total cholesterol, *TG* triglyceride, *LDL-c* low-density lipoprotein cholesterol, *HDL-c* high density lipoprotein cholesterol, *LBP* lipopolysaccharide-binding protein

*Compared with the MUO group, p < 0.05

^#^Compared with the MHO group. Data is expressed either as mean ± SD or median (25th–75th centiles)

The weight, BMI, BMI-Z, WHR, WHtR, SBP, DBP, TG, LDL-c, IL-6, TNF-α, LBP and leptin were significantly higher in the MHO group than the Con children, and HDL-c in the MHO group were significantly lower than the Con group (all p < 0.05). There was no statistical difference in age, gender, FPG and fasting TC between MHO and Con (all p > 0.05, Table 1).

### Microbiota profiles in different metabolic status subjects

A total of 918,578 sequencing reads were obtained from 86 fecal samples, with an average value of 10,681 counts per sample. We identified an overall of 146 OTUs, among which 136 OTU with ≥ 2 counts, and they were grouped in 9 phylum and 38 families.

#### Abundance profiling in different metabolic status subjects

**Fig. 1:**
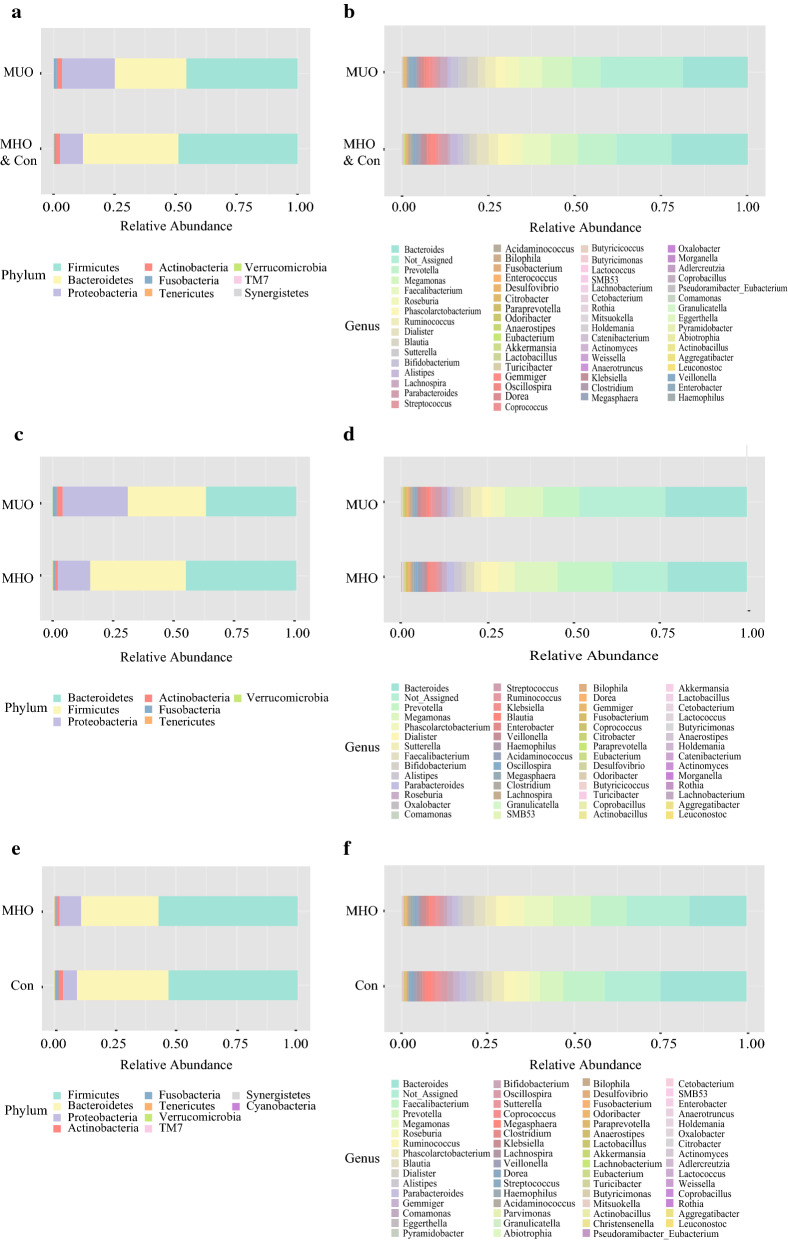
Bar chart representing Mann–Whitney U-test results on operational taxonomic units (OTUs) grouped in phyla (**a**, **c**, **e**) and in genus (**b**, **d**, **f**) of the different metabolic status groups. Each column in the plot represents a group, and each color in the column represents the percentage of relative abundance for each OTU. *MUO* metabolic unhealthy obese, *MHO* metabolically healthy obese, *Con* controls

Grouping OTUs at phylum level, and applying the Mann–Whitney U test on the relative abundances of phyla for the two groups, the relative abundances of phylum *Tenericutes* was more prevalent in the metabolically healthy cohorts (Con and MHO children) compared to the MUO group (p = 0.006, Additional file 1: Table S1 and Fig. 1a).

On OTUs at the genera level, by Mann–Whitney U-test, including all the genera (merging small taxa with counts < 10), we identified that genera *Anaerostipes*, *Alistipes*, *Desulfovibrio*, *Fusobacterium*, *Gemmiger*, *Odoribacter*, *Oscillospira* and *Parabacteroides* were more prevalent in the metabolically healthy cohorts (Con and MHO children) versus MUO children, yet the genus *Dorea* was more prevalent in MUO (*p* < 0.05; Fig. 1b, Table 2).

**Table 2:** The mean relative abundance of gut microbiota with significantly differences in different metabolic status at genera level

	MUO	MHO and Con	Z	P value
*Anaerostipes*	0.001	0.001	− 2.084	0.037
*Odoribacter*	0.000	0.002	− 2.122	0.034
*Desulfovibrio*	0.000	0.003	− 2.142	0.032
*Alistipes*	0.010	0.023	− 2.182	0.029
*Fusobacterium*	0.001	0.002	− 2.185	0.029
*Dorea*	0.012	0.005	− 2.288	0.022
*Gemmiger*	0.007	0.013	− 2.320	0.020
*Oscillospira*	0.008	0.010	− 2.445	0.014
*Parabacteroides*	0.007	0.020	− 2.552	0.011

*MUO* metabolic unhealthy obese, *MHO* metabolically healthy obese, *Con* controls

#### Alpha- and beta-diversity in different metabolic status subjects

**Fig. 2:**
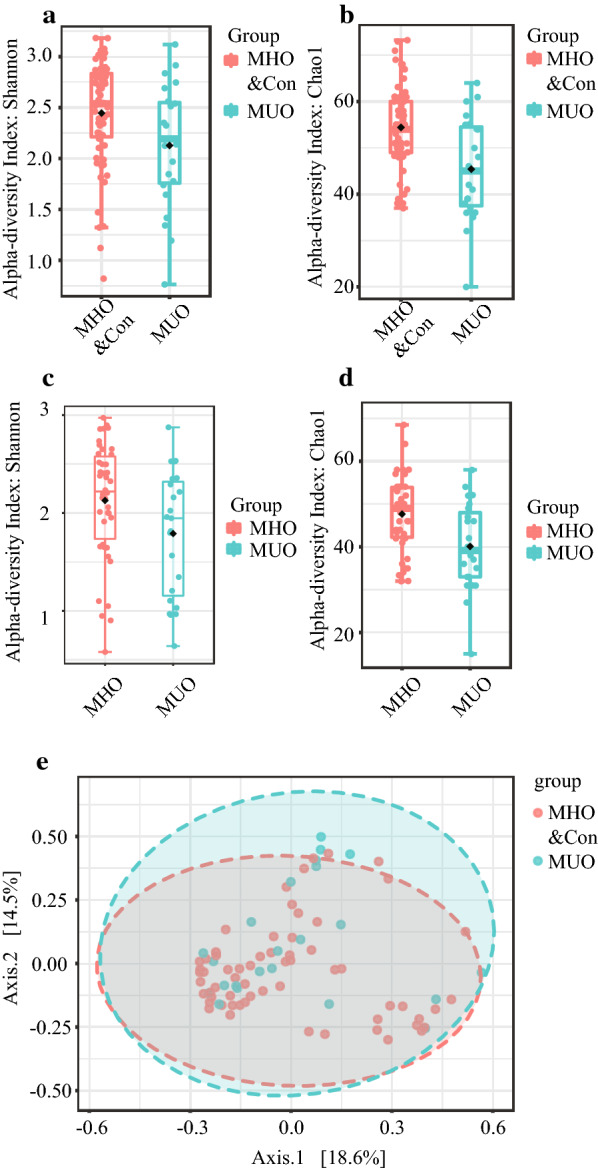
Characterization of alpha- and beta-diversity of the gut microbiota in Con, MUO and MHO groups. The y-axes show the Shannon index (**a**, **c**) and Chao1 richness index (**b**, **d**). The x-axes show the phenotypic categories. Additional data are in Additional file 1: Table S2. Principal coordinates analysis (PCoA) plot of Con and MHO children and MUO subjects (**e**). The plots show the first two principal coordinates (axes) for PCoA using Bray–Curtis Distance method. *MUO* metabolic unhealthy obese, *MHO* metabolically healthy obese, *Con* controls

To assess the overall differences of microbial community structures in metabolic healthy and MUO subjects, we measured ecological parameters based on alpha-diversity. The alpha-diversity analysis showed significantly higher diversity in metabolic healthy subjects (Con and MHO children) in comparison to MUO participants (p < 0.05, Fig. 2a, b, Additional file 1: Table S2).

To determine the differences between microbial community profiles in metabolic healthy and MUO subjects, we calculated beta-diversity. By Distance method Bray–Curtis dissimilarities PCoA analysis, the gut microbiota samples from Con and MHO children were clustered together and separated partly from the MUO group. Upon analysis, the first coordinate (Axis.1) explained the 18.6% of the inter sample variance the second coordinate (Axis.2) explained the 14.5% of the inter sample variance in metabolic healthy subjects (Con and MHO children) in comparison to MUO participants (P = 0.038, Fig. 2e, Additional file 1: Table S3).

#### Bacterial taxa differences in different metabolic status subjects

**Fig. 3:**
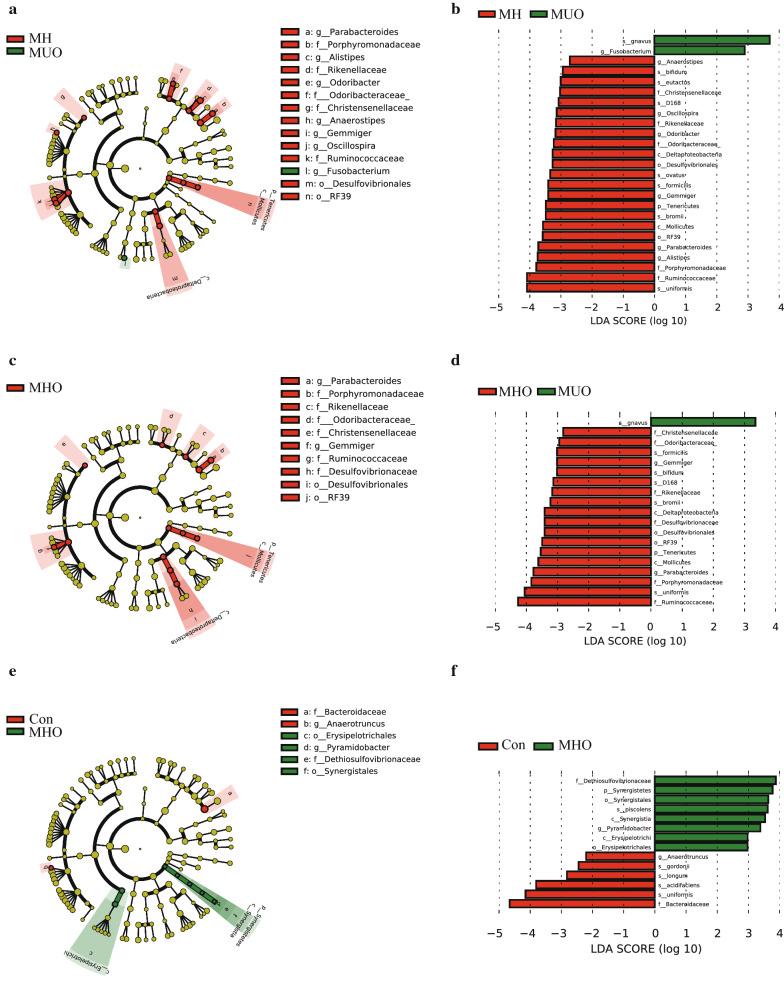
Differential biomarkers associated with different metabolic status. A linear discriminant effect size (LeFse) analysis have been performed (α value = 0.05, logarithmic LDA score threshold = 2.0). *MUO* metabolic unhealthy obese, *MHO* metabolically healthy obese, *Con* controls

We next used LEfSe analysis to identify bacteria in which the relative abundance was significantly increased or decreased in each phenotypic category. The Con and MHO children had members of the phylum *Tenericutes*, class *Deltaproteobacteria*, *Mollicutes*, order *Desulfovibrionales*, *RF39*, family *Christensenellaceae*, *Odoribacteraceae*, *Porphyromonadaceae*, *Ruminococcaceae*, genera *Anaerostipes*, *Oscillospira*, *Odoribacter*, *Gemmiger*, *Parabacteroides*, *Alistipes*, that were significantly higher than MUO subjects. Furthermore, the MUO subjects had members of the genus *Fusobacterium* that were significantly higher than the Con and MHO children (all p < 0.05, Fig. 3a, b).

### Microbiota profiles in obese children with different metabolic status

#### Abundance profiling

Grouping OTUs at phylum level, and applying the Mann–Whitney U test on the relative abundances of phyla for the MHO and MUO groups, the relative abundance of phylum *Tenericutes* was more prevalent in the MHO group compared to the MUO group (p = 0.027, Table 3 and Fig. 1c).

**Table 3:** The mean relative abundance of gut microbiota obese subjects with different metabolic status at phylum level

	MHO	MUO	z	p value
*Actinobacteria*	0.012	0.025	− 0.783	0.434
*Bacteroidetes*	0.453	0.371	− 0.823	0.410
*Firmicutes*	0.393	0.321	− 0.919	0.358
*Fusobacteria*	0.006	0.016	− 1.494	0.135
*Proteobacteria*	0.132	0.267	− 0.535	0.593
*Tenericutes*	0.003	0.000	*− 2.212*	*0.027*
*Verrucomicrobia*	0.001	0.000	− 1.480	0.139

*MHO*, metabolically healthy obese;* MUO*: metabolic unhealthy obese

Italicized value * P* < 0.05

On OTUs at the genera level, by Mann–Whitney U analysis, including all the genera (merging small taxa with counts < 10), we identified that genera *Desulfovibrio*, *Parabacteroides* and *Gemmiger* were more prevalent in MHO subjects compared to MUO subjects (*p* = 0.027, 0.040 and 0.047, respectively; Fig. 1d).

#### Alpha- and beta-diversity between MHO and MUO subjects

Regarding alpha-diversity, in both the MHO and MUO group, the analysis exposed significantly higher diversity in MHO subjects versus MUO participants (all p < 0.05, Fig. 2c, d, Additional file 1: Table S2).

Regarding beta-diversity, by an unweighted-UniFrac method, the MHO group was lower than the MUO group (p = 0.021, Additional file 1: Table S3).

#### Bacterial taxa differences between MHO and MUO subjects

LEfSe analysis showed MHO subjects had members of the phylum *Tenericutes*, *class Deltaproteobacteria*, *Mollicutes*, order *Desulfovibrionales*, *RF39*, family *Christensenellaceae*, *Odoribacteraceae*, *Rikenellaceae*, *Desulfovibrionaceae*, *Porphyromonadaceae*, *Ruminococcaceae*, genus *Gemmiger*, *Parabacteroides* that were significantly higher than MUO subjects (all p < 0.05, Fig. 3c, d).

### Microbiota profiles in MHO and Con children with different weight status

#### Abundance profiling

Grouping OTUs at phylum level, the relative abundances of phylum *Synergistetes* was more prevalent in the MHO group compared to the Con group (p < 0.05, Fig. 1e, Table 4).

**Table 4:** The mean relative abundance of gut microbiota with significantly differences in obese subjects with different metabolic status at genera level

	MHO	Con	Z	P value
*Actinobacteria*	0.012	0.018	− 1.181	0.238
*Bacteroidetes*	0.319	0.377	− 1.006	0.314
*Cyanobacteria*	0.000	0.000	− 1.245	0.213
*Firmicutes*	0.572	0.531	− 0.831	0.406
*Fusobacteria*	0.006	0.014	− 0.324	0.746
*Proteobacteria*	0.088	0.057	− 1.881	0.060
*Synergistetes*	0.000	0.000	− 1.964	*0.050*
*Tenericutes*	0.002	0.002	− 1.408	0.159
*TM7*	0.000	0.000	− 0.481	0.630
*Verrucomicrobia*	0.001	0.001	−0.177	0.859

*MHO*, metabolically healthy obese;* Con*, control

Italicized value* P* < 0.05

On OTUs at the genera level, including all the genera (merging small taxa with counts < 10), genera *Anaerotruncus*, *Bacteroides*, *Adlercreutzia* and *Pyramidobacter* were more prevalent in MHO subjects versus MUO subjects (*p* < 0.05; Fig. 1f).

#### Alpha- and beta-diversity between different weight status

Regarding alpha-diversity, the Shannon diversity index, Observed OTUs, Faith’s phylogenetic diversity and Pielou’s evenness based on OTU distribution did not reveal any significant difference between MHO and Con (all p > 0.05, Additional file 1: Table S2); also, beta-diversity did not differ significantly between these two groups. Importantly, none of the comparisons were significantly different (all p > 0.05) after correction for multiple testing (Additional file 1: Table S3).

#### Bacterial taxa differences in MHO and Con children of different weight status

LEfSe analysis showed MHO subjects had members of the phylum *Synergistetes*, class *Synergistia*, order *Synergistales*, *Erysipetotrichales*, family *Dethiosulfovibrionaceae*, genus *Pyramidobacter* were significantly higher than the Con-, however, the latter had members of the family *Bacteroidaceae*, genus *Anaerotruncus* that were significantly higher (all p < 0.05, Fig. 3e, f).

### Correlations between inflammatory factors and bacterial abundance

To evaluate correlations between bacteria and serum inflammatory factors (IL-6, TNF-α and leptin), Spearman’s rho cut-off values were assessed, taking into account r > 0.4, r < − 0.4 (p < 0.05, Additional file 1: Table S4).

For MUO subjects, Spearman’s correlation analysis revealed that IL-6 positively correlated with genus *Lactococcus*, TNF-α positively correlated with phylum *Bacteroidetes*, negatively correlated with genus *Citrobacter*. LBP positively correlated with genus *Prevotella*, *Odoribacter*, and negatively correlated with genus *Bifidobacterium*, *Streptococcus*, *Roseburia*, *Clostridium* and *Veillonella.* Leptin positively correlated with genus *Eubacterium* and negatively correlated with genus *Faecalibacterium* and *Lachnospira* (all p < 0.05, Additional file 1: Table S4).

For MHO subjects, Spearman’s correlation analysis revealed that serum IL-6 positively correlated with genus *Paraprevotella*. LBP positively correlated with genus *Roseburia* and *Faecalibacterium*, and negatively correlated with genus *Lactobacillus.* Leptin positively correlated with phylum *Bacteroidetes*, *Firmicutes*, genus *Phascolarctobacterium* and negatively correlated with genus *Dialister* (all p < 0.05). There was no association between the bacteria and TNF α at the genus level (all p > 0.05).

### Metabolic pathway predictions

**Fig. 4:**
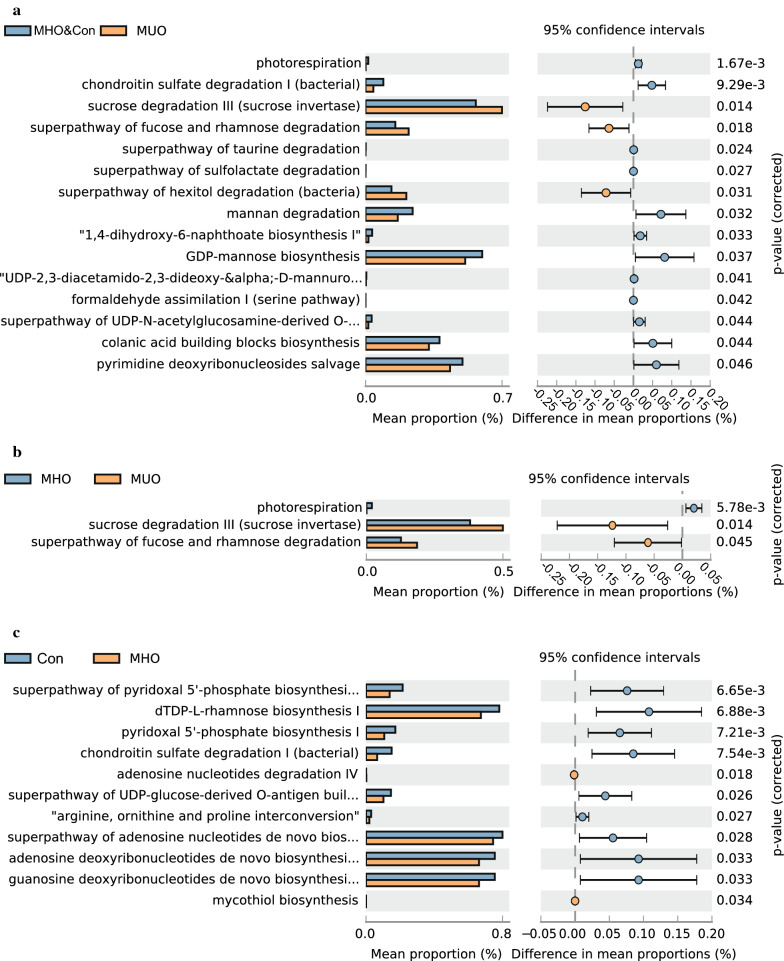
KEGGs biomarkers associated with the three metabolic statuses. *MUO* metabolic unhealthy obese, *MHO* metabolically healthy obese, *Con* controls

A total of 15 KEGG pathways were generated using the composition of the fecal microbiota based on PICRUSt2 in the metabolic healthy cohorts (MHO and Con subjects) versus MUO subjects (Fig. 4, Additional file 1: Table S5). Importantly, the glucose metabolism pathways, including GDP-mannose biosynthesis and superpathway of UDP-*N*-acetylglucosamine-derived O-antigen building blocks biosynthesis, were increased in metabolic healthy cohorts and, conversely, the superpathway of fucose and rhamnose degradation were alternated in the metabolic healthy cohorts (all p < 0.05). In the comparison between MHO and MUO subjects, we obtained 3 differential pathways including superpathway of fucose and rhamnose degradation, photorespiration, and sucrose degradation III, which were also observed significantly different between the metabolic healthy cohorts (MHO and Con subjects) versus MUO subjects (Fig. 4, Additional file 1: Table S6). Moreover, 11 differential metabolic patterns differentially expressed resulted in the comparison between MHO versus Con (Fig. 4, Additional file 1: Table S7).

## Discussion

Recognized for decades, there is wide-ranging heterogeneity among obese individuals as to their risk for developing metabolic dysfunction and its attendant complications [12]. Also well-established, and which may contribute to this metabolic heterogeneity, is the fact those with central obesity are more prone to developing T2DM and cardiovascular disease than those with peripheral obesity [13]. In this study, to indirectly address the issue of fat distribution, we found there were no significant differences in WHR and WHtR between the two obese cohorts, MHO vs. MUO.

A chronic low-grade inflammation, triggered by nutrient surplus, is a constituent of obesity. Adipose-originated metabolic inflammation develops pari passu with insulin resistance and, as such, is a key element in the metabolic syndrome [14]. In this study, we found there were no significant differences in serum IL-6, TNF-α, LBP and leptin between MHO and MUO subjects. It stands to reason that, besides these cytokines, other biochemical factors likely contribute to the metabolic diverseness in obese subjects. Or, perhaps, the concentrations of circulating compounds—such as those abovementioned—poorly reflect those found in extracellular or intracellular tissues.

Evidence can be adduced that the gut microbiota is involved in the aetiology of obesity and obesity-related complications such as nonalcoholic fatty liver disease, insulin resistance and T2DM [15, 16]. These disorders are characterized by alterations in the diversity of the gut microbiota, and the relative abundance of certain genera. And bacteria-generated metabolites, translocated from the gut across a disrupted intestinal barrier, can affect several metabolic organs, such as the liver and adipose, thereby contributing to systemic metabolic inflammation [17].

Recently, several animal studies concluded that an optimal healthy-like gut microbiota may bestow a more propitious obese phenotype [18, 19]. For instance, the abundance of *Bacteroidetes* and *Tenericutes* were closely aligned with bile acid metabolism and obesity-related inflammation in a murine model of the metabolic syndrome [20]. In our study, we corroborate this finding: reduced abundance of *Tenericutes* in the MUO group compared with the metabolically healthy groups (MHO and Con). Moreover, individuals with diminished insulin sensitivity had lower abundance of *Tenericutes* [21]. And, in animal experiments, administration of hydrogenated xanthohumol, which mitigates the metabolic syndrome by altering gut microbiota diversity and abundance, specifically, a reduction in *Bacteroidetes* and *Tenericutes* [20]. These results suggested an important role of *Tenericutes* in metabolism. We also observed greater abundance of *Anaerostipes* in the MHO and Con cohort, as well as the alpha and beta diversity. Using separate-sample Mendelian randomization to obtain estimates of the associations of 27 genera of gut microbiota with cardiovascular disease risks, *Anaerostipes* was identified as being nominally associated with T2DM [22], and this effect may be a result of butyrate production [23]. These results buttress the notion of dysbiosis in the gut microbiota of MUO individuals.

To characterize the gut microbiota in obese children of different metabolic status, we further analyze the MHO and the MUO groups. The abundance of *Tenericutes* was significantly reduced in the MUO group compared with the metabolic healthy children, indicating that *Tenericutes* is related to the metabolic state, and the bacterial imbalance is independent of weight. Previously reported, the abundance of *Parabacteroides* was significantly decreased in obese subjects with metabolic syndrome [6], and nonalcoholic fatty liver disease [24], and negatively correlated with weight gain and leptin plasma levels [25]. And germane to our findings, both genera *Gemmiger* [26] and *Parabacteroides* [27] are gut bacteria negatively associated with obesity and disturbed host metabolism. In accordance, we found that that the fecal abundance of these bacteria was significantly higher in the MHO group compared with MUO.

The genera *Parabacteroides* are short-chain fatty acids (SCFAs)-producing bacteria. SCFAs are low molecular weight molecules produced from fermentation of dietary fiber or polysaccharides by gut microbiota. Absorbed by the intestinal epithelium into the blood, they can beget physiological disorders in the host, such as deranged lipid metabolism and intestinal environment imbalances [28, 29]. In our determination, alpha and beta diversity were significantly higher in Con and MHO children compared with the MUO group, again supporting the notion of dysbiosis in the unhealthy MUO population.

Notwithstanding that the gut microbiota of obese individuals with metabolic syndrome may indeed be unhealthy, is the gut microbiota of the MHO population really healthy? We compared the characteristic of gut microbiota in the Con and MHO children of different weights. Even though there was no significant difference in alpha and beta diversity, the relative abundances of phylum *Synergistetes* and genus *Bacteroides* were elevated in the MHO group compared to the Con children. Based on a metagenomic approach and bioinformatics analysis in obese adults, it is plausible that an abundance of the microbiota taxa *Bacteroides* could portent the evolution to T2DM [30].

Alterations in gut ecology can propel inflammatory pathways in several tissues, resulting in glucose intolerance and CVD [31, 32]. In rodents, a disturbance in the tripartite interactions between the microbiota, bile acids, and host metabolism, along with the bacterial production of lipopolysaccharides (LPS, i.e., endotoxemia), can beget derangements in glucose homeostasis [16, 26]. LBP is an acute inflammation phase protein that complexes with LPS and facilitates binding with CD14. In adolescents, serum LBP robustly correlates positively with indices of abnormal glucose and lipid metabolism. Herein, we found that, depending on the metabolic status, the serum levels of classic proinflammatory factors IL-6, TNF-α, LBP and leptin were related to the abundance of various fecal bacteria. Notably, in MHO children, serum leptin correlated positively with genus *Phascolarctobacterium* and negatively with *Dialister*—the latter genera observed with low abundance in obese children [33]. And, relevant to our findings, it is noteworthy that *Phascolarctobacterium* is purportedly a biomarker for adult T2DM [30]. In high fat diet obese mice with insulin resistance, *Prevotella* was deemed as pro-inflammatory and, of note, its abundance in our study correlated with serum LBP [34]*.* As illustrated in our MHO children and the above-cited studies in humans, the gut microbiota is a marquee player in preserving normal metabolism despite obesity or, perhaps, an ephemeral protective microbiota destined to change with transition to MUO.

Compared to the metabolic healthy cohorts in the MUO children, several pathways associated with glucose and lipid metabolism pathways, such as fucose and rhamnose degradation and sucrose degradation III were increased. Conversely, mannan degradation was markedly decreased. Of interest, serum fucose levels are higher in the T2DM patients compared to healthy cohorts [35]. Mannan-oligosaccharide in the diet improves the metabolic syndrome in mice, alternatively insulin resistance and dyslipidemia [36, 37]. We found that bacterial fucose and rhamnose degradation and sucrose degradation III were increased in the MUO subjects compared with the MHO subjects, inferring that the change was independent of weight. However, insofar as serum levels of fucose were undetectable, and the dietary intake of sucrose and mannan were not assessed in our study, future longitudinal studies could conceivably unravel the intricate, possibly causual, relationships between the gut microbiota, obesity, and aberrant intermediary host metabolism.

## Conclusion

In aggregate, the MUO population had lower alpha- and beta-diversity, and lower abundance of *Tenericutes*, inferring a robust intricate inter-relationship between gut bacterial ecology and host metabolic state. In the MHO population, phylum *Synergistetes* and genus *Bacteroides* and *Phascolarctobacterium* were more prevalent, and the abundance of some metabolism-related bacteria correlated with circulating proinflammatory factors, suggesting that compared to healthy controls, dysbiosis of gut microbiota was already extant in the MHO children, and conceivably a compensatory or remedial response to a surfeit of nutrients.

## Methods

### Study population

This study was approved by the Ethics Committee of the Fuzhou Children’s Hospital of Fujian Medical University and, in all cases, informed consent was obtained.

The cross-sectional study consisted of participants managed by Fuzhou Children’s Hospital of Fujian Medical University from September 2017 to March 2018. This study was limited to participants who met the following criteria: (a) ages between 5 to 15 years old, and (b) residence of Fujian province.

The exclusion criteria were as follows: any endocrine disorder, history of antibiotic therapy in the past 3 months prior to the enrollment, chronic gastrointestinal illness or use of gastro-intestinal-related medication, or diarrheal disease (World Health Organization definition) in the past 1 month.

### Clinical assessment

Height and weight were measured by trained nurses. BMI-Z scores were calculated based on reference values of Li et al. [38]. At the end of normal expiration, waist and hip circumference were measured to the nearest 0.5 cm using standard technique with nonelastic tape. Waist circumference was measured at a point midway between the lower border of the ribs and the iliac crest, and hip circumference was measured at the widest part of the hip. A waist-to-hip ratio (WHR) was calculated by waist circumference (cm) divided by hip circumference (cm) and a waist-to-height ratio (WHtR) by waist circumference (cm) divided by height (cm).

### Laboratory methods

All participants maintained their usual dietary pattern at least 3 days before blood sampling. After 12 h of fasting, 10 mL venous blood was drawn by registered nurses. All blood samples were stored at − 80 ℃, and analyzed within two weeks of sampling. Serum IL-6 was measured using a commercial ELISA kit (Abcam, UK), with an 4.4% inter-assay coefficient of variation (CV). Serum TNF-α levels was measured using a commercial ELISA kit (Abcam, UK), with inter-assay and intra-assay CVs of 3.3% and 9%, respectively, and serum leptin assayed using a commercial ELISA kit (Abcam, UK), with inter-assay and intra-assay CVs of 2.4% and 2.7%, respectively. The serum LBP levels were measured using a commercial ELISA kit (Abnova, Taiwan, China), with inter-assay and intra-assay CV 9.8–17.8% and 6.1%, respectively. Fasting plasma glucose (FPG) and plasma lipids, including total cholesterol (TC), triglyceride (TG), high-density lipoprotein cholesterol (HDL-c) and low density lipoprotein cholesterol (LDL-c), were assayed by standard methods using specific reagents (Beckman Coulter AU5800, USA). Fasting insulin (INS) was determined by a chemiluminescent immunoassay (IMMULITE 2000, Siemens Healthcare Diagnostics Products Limited, Germany). Fecal samples were collected and processed as previously described [39].

### Definition of metabolic unhealthy

Metabolic syndrome parameters were applied according to 2019 Expert Committees [40], and MUO was defined by the presence of at least one of the following metabolic traits: (1) FPG ≥ 5.6 mmol/L; (2) systolic blood pressure ≥ 90th percentile for gender and age; (3) fasting HDL-C < 1.03 mmol/L; and (4) fasting TG ≥ 1.7 mmol/L.

### Genomic DNA extraction and library construction

The microbial community DNA was extracted and quantified as previously described [39]. Variable regions V3–V4 of bacterial 16s rRNA gene were amplified with degenerate PCR primers [39]. Libraries were qualified by the Agilent 2100 bioanalyzer (Agilent, USA). The validated libraries were used for sequencing on Illumina MiSeq platform (BGI, Shenzhen, China) following the standard pipeline of Illumina, and generating 2 × 300 bp paired-end reads.

### Statistical analysis

Statistical analyses of clinical data were performed using the Statistical Package for the Social Sciences software version 23.0 (SPSS Inc. Chicago, IL, USA). The normality of the data was tested by Kolmogorov–Smirnov test. Data are expressed as mean ± SD or median (25th–75th percentiles). Comparisons of the results were assessed using independent samples t test, Mann–Whitney U test and Kruskal–Wallis test, depending on the type of data distribution (e.g., non parametric). Comparison of rates between two groups was by chi-square. A value of *P* < 0.05 was deemed statistically significant.

Statistical analysis of 16s rRNA sequencing data were performed on alpha- and beta-diversity measurements, which was done by software QIIME2 (v2019.7) [41]. Kruskal–Wallis Test was adopted for two groups comparison. Linear discriminant analysis Effect Size (LEfSe) Analysis was assessed by software LEFSE [42]. To predict metagenome functional content from 16S rRNA gene surveys, Picrust2 [43] have been applied to obtain the Kyoto Encyclopedia of Genes and Genomes (KEGG) pathways, and STAMP [44] was used to analyze the differential pathways.

## Supplementary Information

**Additional file 1: Table S1.** The mean relative abundance of gut microbiota in different metabolic status at phylum level. **Table S2.** Comparison of alpha-diversity in obese subjects with different metabolic status. **Table S3.** Comparison of beta-diversity between different metabolic status. **Table S4.** Spearman’s correlation table on OTUs and inflammatory factors in MHO and MUO groups. **Table S5.** KEGGs biomarkers in MHO and Con subjects compared with MUO subjects. **Table S6.** KEGGs biomarkers in MHO and MUO subjects. **Table S7.** KEGGs biomarkers in MHO and Con subjects.

## Data Availability

The original contributions presented in the study are publicly available. The raw sequence data reported in this paper have been deposited in the Genome Sequence Archive (Genomics, Proteomics & Bioinformatics 2017) in National Genomics Data Center (Nucleic Acids Res 2020), Beijing Institute of Genomics (China National Center for Bioinformation), Chinese Academy of Sciences, under accession number CRA003010 that are publicly accessible at https://bigd.big.ac.cn/gsa.
