# Supplementary material for: The role of the gut microbiota on the metabolic status of obese children
Source: Microb Cell Fact. 2021 Feb 27;20:53. doi: 10.1186/s12934-021-01548-9 (PMC7916301; doi:10.1186/s12934-021-01548-9)
Supplement: Supplementary file 1 — Additional file 1: Table S1. The mean relative abundance of gut microbiota in different metabolic status at phylum level. Table S2. Comparison of alpha-diversity in obese subjects with different metabolic status. Table S3. Comparison of beta-diversity between different metabolic status. Table S4. Spearman’s correlation table on OTUs and inflammatory factors in MHO and MUO groups. Table S5. KEGGs biomarkers in MHO and Con subjects compared with MUO subjects. Table S6. KEGGs biomarkers in MHO and MUO subjects. Table S7. KEGGs biomarkers in MHO and Con subjects. [file 12934_2021_1548_MOESM1_ESM.docx]

**Additional file 1**

Table S1. The mean relative abundance of gut microbiota in different metabolic status at phylum level

|  | MUO | MHO and Con | z | p value |
| --- | --- | --- | --- | --- |
| *Actinobacteria* | 0.022 | 0.017 | -1.327 | 0.184 |
| *Bacteroidetes* | 0.294 | 0.392 | -1.634 | 0.102 |
| *Firmicutes* | 0.455 | 0.488 | -0.327 | 0.744 |
| *Fusobacteria* | 0.013 | 0.007 | -1.673 | 0.094 |
| *Proteobacteria* | 0.215 | 0.093 | -0.863 | 0.388 |
| *Synergistetes* | 0.000 | 0.000 | -0.71 | 0.478 |
| ***Tenericutes*** | **0.000** | **0.002** | **-2.743** | **0.006** |
| *TM7* | 0.000 | 0.000 | -0.994 | 0.320 |
| *Verrucomicrobia* | 0.000 | 0.001 | -1.74 | 0.082 |

MUO: metabolic unhealthy obese; MHO: metabolically healthy obese; Con: controls;

Table S2 Comparison of alpha-diversity in obese subjects with different metabolic status

| groups | alpha-diversity index | H | P value |
| --- | --- | --- | --- |
| MHO and Con vs MUO | Shannon | **5.369** | **0.020** |
|  | Observed OTUs | **10.494** | **0.001** |
|  | Faith’s phylogenetic diversity | **12.371** | **<0.001** |
|  | Pielou’s evenness | 2.185 | 0.139 |
| MHO vs MUO | Shannon | **4.067** | **0.044** |
|  | Observed OTUs | **5.128** | **0.023** |
|  | Faith’s phylogenetic diversity | **7.987** | **0.005** |
|  | Pielou’s evenness | 3.795 | 0.051 |
| MHO vs Con | Shannon | 0.691 | 0.406 |
|  | Observed OTUs | 0.232 | 0.630 |
|  | Faith’s phylogenetic diversity | 0.155 | 0.694 |
|  | Pielou’s evenness | 0.871 | 0.351 |

MUO: metabolic unhealthy obese; MHO: metabolically healthy obese; Con: controls.

Table S3 Comparison of beta-diversity between different metabolic status

| groups | beta-diversity index | pseudo-F | P value |
| --- | --- | --- | --- |
| MHO and Con vs MUO | Bray-Curtis distance | 1.314 | 0.053 |
|  | Jaccard distance | **1.438** | **0.002** |
|  | Unweighted-unifrac | **2.893** | **0.002** |
|  | Weighted-unifrac | 2.121 | 0.058 |
| MHO vs MUO | Bray-Curtis distance | 1.114 | 0.260 |
|  | Jaccard diatance | 1.109 | 0.088 |
|  | Unweighted-unifrac | **1.888** | **0.021** |
|  | Weighted-unifrac | 1.105 | 0.327 |
| MHO vs Con | Bray-Curtis distance | 1.211 | 0.135 |
|  | Jaccard diatance | 1.132 | 0.136 |
|  | Unweighted-unifrac | 1.046 | 0.306 |
|  | Weighted-unifrac | 0.757 | 0.571 |

MUO: metabolic unhealthy obese; MHO: metabolically healthy obese; Con: controls;

Table S4 Spearman’s correlation table on OTUs and inflammatory factors in MHO and MUO groups.

|  | MUO | | | | MHO | | | |
| --- | --- | --- | --- | --- | --- | --- | --- | --- |
|  | IL-6 | TNF-α | LBP | Leptin | IL-6 | TNF-α | LBP | Leptin |
| IL-6 | 1 | .453* | 0.3 | -0.42 | 1 | 0.166 | 0.128 | 0.057 |
| TNF-α | .453* | 1 | 0.258 | -0.14 | 0.166 | 1 | 0.045 | -0.05 |
| LBP | 0.3 | 0.258 | 1 | 0.057 | 0.128 | 0.045 | 1 | 0.138 |
| Leptin | -0.42 | -0.14 | 0.057 | 1 | 0.057 | -0.05 | 0.138 | 1 |
| g__Actinomyces | 0.019 | 0.111 | -0.24 | 0.364 | -0.18 | -0.04 | 0.01 | 0.035 |
| g__Rothia | 0.101 | -0.03 | -0.36 | 0.25 | -0.12 | 0.03 | 0.212 | 0.041 |
| g__Bifidobacterium | -0.11 | 0.061 | -.600** | 0.156 | 0.096 | -0.06 | -0.25 | 0.235 |
| g__Adlercreutzia | -0.15 | -0.15 | -0.22 | 0.332 | 0.194 | 0.125 | 0.009 | 0.101 |
| g__Eggerthella | 0.13 | 0.252 | -0.04 | 0.296 | -0.21 | -0.12 | -0.04 | 0.134 |
| g__Bacteroides | -0 | 0.129 | 0.073 | -0.42 | -0.07 | 0.058 | 0.205 | -0.14 |
| g__Parabacteroides | 0.099 | 0.407 | 0.277 | 0.009 | 0.228 | 0.157 | -0.04 | -0.02 |
| g__Prevotella | -0.19 | -0.02 | .438* | 0.366 | 0.196 | -0.06 | -0.02 | -0.13 |
| g__Alistipes | -0.17 | 0.107 | 0.129 | 0.076 | 0.272 | 0.189 | -0.02 | -0.12 |
| g__human | . | . | . | . | -0.16 | -0.19 | 0.135 | 0.135 |
| g__Barnesiella | . | . | . | . | 0.213 | 0.264 | -0.06 | 0.071 |
| g__Butyricimonas | -0.09 | -0.17 | 0.011 | 0.04 | 0.073 | -0.18 | -0.05 | -0.03 |
| g__Odoribacter | 0.007 | 0.343 | .446* | 0.213 | 0.016 | 0.071 | -0.07 | -0.1 |
| g__Paraprevotella | -0.18 | 0.095 | 0.098 | 0.402 | .319^*^ | -0.03 | 0.088 | 0.068 |
| g__[Prevotella] | -0.26 | 0.148 | 0.369 | 0.295 | -0.07 | -0.06 | 0.119 | 0.175 |
| g__Abiotrophia | 0.022 | 0.427 | -0.25 | 0.225 | -0.06 | 0.226 | 0.032 | 0.251 |
| g__Granulicatella | 0.151 | 0.053 | -0.21 | 0.272 | -0.2 | 0.042 | 0.286 | 0.179 |
| g__Enterococcus | 0.185 | 0.074 | -0.07 | 0.148 |  |  |  |  |
| g__Lactobacillus | -0.08 | -0.04 | -0.21 | 0.239 | 0.244 | -0.14 | -.363^*^ | 0.069 |
| g__Leuconostoc | . | . | . | . | -0.12 | 0.077 | -0.03 | 0.246 |
| g__Weissella | 0.222 | -0.02 | -0.05 | 0.069 | 0.202 | -0.08 | -0.09 | 0.048 |
| g__Lactococcus | .451* | -0.07 | 0.183 | -0.19 | 0.256 | -0.03 | -0.15 | -0.01 |
| g__Streptococcus | 0.165 | 0.201 | -.473* | 0.172 | -0.1 | -0.06 | -0.27 | 0.162 |
| g__Turicibacter | -0.33 | -0.38 | -0.22 | 0.209 | -0.04 | 0.05 | 0.037 | 0.258 |
| g__Clostridium | 0.209 | -0.11 | -0.38 | -0.26 | 0.06 | 0.106 | -0.26 | 0.217 |
| g__SMB53 | -0.25 | -0.2 | -0.3 | 0.367 | 0.165 | -0.11 | 0.067 | 0.092 |
| g__Pseudoramibacter_Eubacterium | 0.037 | 0.332 | -0.15 | 0.222 | 0.243 | 0.156 | 0.172 | 0.089 |
| g__Anaerostipes | 0.137 | 0.212 | 0.241 | -0.1 | -0.28 | -0.04 | 0.009 | 0.186 |
| g__Blautia | -0.1 | -0.14 | -0.03 | 0.178 | 0.123 | 0.092 | -0.13 | 0.28 |
| g__Clostridium | -0.3 | -0.07 | 0.048 | -0.03 | 0.088 | 0.019 | -0.1 | 0.118 |
| g__Coprococcus | 0.016 | -0.22 | -0.02 | -0.12 | -0.01 | -0.01 | -0.09 | 0.081 |
| g__Dorea | -0.13 | 0.081 | -0.4 | -0.01 | 0.032 | -0.02 | -0.17 | 0.223 |
| g__Lachnobacterium | . | . | . | . | 0.193 | 0.106 | 0.026 | -0.14 |
| g__Lachnospira | 0.02 | -0.14 | -0.18 | -.528* | -0.3 | -0.05 | 0.144 | -0.06 |
| g__Roseburia | -0.27 | 0.042 | -.466* | -0.39 | 0.021 | 0.113 | .488^**^ | 0.145 |
| g__[Ruminococcus] | -0.22 | -0.29 | -0.24 | -0.06 | -0.03 | -0.02 | -0.01 | 0.289 |
| g__[Clostridium] | -0.26 | -0.36 | -.454* | -0.15 | 0.143 | -0.16 | 0.121 | 0.304 |
| g__Anaerotruncus | -0.28 | 0.22 | 0.095 | 0.185 | 0.247 | 0.02 | -0.09 | 0.149 |
| g__Butyricicoccus | 0.054 | 0.21 | -0.13 | -0.34 | 0.022 | -0.14 | -0.09 | -0.21 |
| g__Faecalibacterium | -0.13 | -0.09 | -0.15 | -.565** | -0.21 | -0.14 | .357^*^ | -0.03 |
| g__Gemmiger | 0.081 | 0.004 | 0.143 | 0.017 | -0.05 | -0.02 | -0.24 | 0.157 |
| g__Oscillospira | -0.23 | 0.205 | 0.068 | 0.117 | 0.142 | 0.032 | -0.08 | -0.01 |
| g__Ruminococcus | -0.3 | -0.05 | -0.1 | -0.06 | 0.082 | 0.132 | 0.004 | 0.171 |
| g__Acidaminococcus | 0.074 | -0.04 | 0.258 | -0.26 | 0.187 | 0.244 | -0.16 | 0.082 |
| g__Dialister | -0.13 | 0.049 | -0.03 | 0.058 | -0.22 | -0.03 | -0.08 | -.322^*^ |
| g__Megamonas | -0.22 | 0.248 | 0.003 | 0.34 | 0.295 | -0.07 | 0.193 | -0.05 |
| g__Megasphaera | 0.169 | 0.053 | -0.18 | -0.1 | -0.24 | -0.03 | 0.076 | -0.12 |
| g__Mitsuokella | . | . | . | . | -0.2 | 0.084 | -0.17 | -0.11 |
| g__Phascolarctobacterium | -0.36 | -0.01 | -0.11 | 0.114 | 0.149 | -0.02 | -0.13 | .319^*^ |
| g__Veillonella | -0.15 | 0.068 | -.641** | -0 | -.343^*^ | 0.057 | -0.18 | -0.16 |
| g__Catenibacterium | -0.33 | -0.28 | 0.148 | 0.369 | 0.135 | 0.239 | 0.006 | -0.1 |
| g__Clostridium | 0.038 | 0.044 | -0.3 | 0.274 | 0.034 | 0.012 | 0.006 | 0.205 |
| g__Coprobacillus | -0.13 | -0.32 | 0.134 | 0.214 | 0.039 | -0.23 | -0.09 | -0.01 |
| g__Holdemania | 0.123 | 0.038 | 0.251 | -0.22 | 0.166 | -0.04 | -0.17 | -0.05 |
| g__[Eubacterium] | -0.34 | 0.064 | 0.129 | .649** | 0.151 | -0.01 | 0.042 | -0.03 |
| g__Cetobacterium | . | . | . | . | 0.095 | -0.25 | 0.049 | 0.202 |
| g__Fusobacterium | 0.129 | -0.32 | 0.039 | -0.32 | -0.1 | 0.149 | 0.023 | 0.1 |
| g__Sutterella | -0.39 | -0.1 | -0.27 | -0.19 | -0.13 | -0.08 | -0.1 | -0.04 |
| g__Comamonas | 0.222 | 0.037 | -0.37 | -0.37 | -0.22 | -0.08 | -0.01 | 0.045 |
| g__Oxalobacter | 0.112 | 0.337 | 0.36 | 0.18 | 0.252 | 0.043 | -0.08 | 0.101 |
| g__Bilophila | -0.25 | 0.11 | -0.18 | 0.171 | 0.065 | 0.142 | 0.082 | -0.15 |
| g__Desulfovibrio | -0.17 | -0.11 | -0.02 | -0.21 | 0.054 | -0.01 | 0.056 | 0.073 |
| g__Citrobacter | -0.24 | -.602** | -0.28 | 0.11 | 0.117 | 0.146 | 0.07 | -0.03 |
| g__Enterobacter | 0.063 | -0.39 | 0.041 | -0.08 | 0.222 | 0.083 | -0.13 | 0.04 |
| g__Klebsiella | 0.05 | -0.09 | -0.4 | -0.11 | 0.202 | -0.05 | -0.16 | -0 |
| g__Morganella | 0.148 | 0 | 0.111 | 0.185 | -0.22 | -0.08 | -0.24 | -0.02 |
| g__Actinobacillus | -0.33 | -0.28 | 0.148 | 0.369 | -0.05 | -0.15 | 0.295 | 0.249 |
| g__Aggregatibacter | . | . | . | . | 0.157 | -0.15 | 0.022 | 0.198 |
| g__Haemophilus | 0.085 | -0.08 | -0.11 | -0.3 | -0.13 | 0.131 | 0.249 | 0.205 |
| g__Pyramidobacter | 0.332 | 0.185 | 0.332 | -0.07 | 0.282 | 0.106 | 0.016 | 0.149 |
| g__Akkermansia | 0.135 | 0.143 | 0.094 | -0.12 | 0.003 | 0.048 | -0.22 | 0.277 |

MUO: metabolic unhealthy obese; MHO: metabolically healthy obese; *:P<0.05; **:p <0.01

Table S5 KEGGs biomarkers in MHO and Con subjects compared with MUO subjects.

| pathway | 1: mean rel. freq. (%) | 1: std. dev. (%) | 2: mean rel. freq. (%) | 2: std. dev. (%) | p-values |
| --- | --- | --- | --- | --- | --- |
| colanic acid building blocks biosynthesis | 0.35 | 0.10 | 0.30 | 0.10 | 0.04 |
| superpathway of fucose and rhamnose degradation | 0.14 | 0.08 | 0.21 | 0.11 | 0.02 |
| superpathway of hexitol degradation (bacteria) | 0.12 | 0.10 | 0.20 | 0.14 | 0.03 |
| superpathway of taurine degradation | 0.00 | 0.00 | 0.00 | 0.00 | 0.02 |
| formaldehyde assimilation I (serine pathway) | 0.00 | 0.00 | 0.00 | 0.00 | 0.04 |
| photorespiration | 0.01 | 0.03 | 0.00 | 0.00 | 0.00 |
| GDP-mannose biosynthesis | 0.56 | 0.13 | 0.48 | 0.16 | 0.04 |
| sucrose degradation III (sucrose invertase) | 0.53 | 0.19 | 0.65 | 0.20 | 0.01 |
| chondroitin sulfate degradation I (bacterial) | 0.09 | 0.10 | 0.04 | 0.06 | 0.01 |
| superpathway of sulfolactate degradation | 0.00 | 0.00 | 0.00 | 0.00 | 0.03 |
| UDP-2,3-diacetamido-2,3-dideoxy-&alpha;-D-mannuronate biosynthesis | 0.00 | 0.00 | 0.00 | 0.00 | 0.04 |
| pyrimidine deoxyribonucleosides salvage | 0.46 | 0.12 | 0.40 | 0.12 | 0.05 |
| superpathway of UDP-N-acetylglucosamine-derived O-antigen building blocks biosynthesis | 0.03 | 0.04 | 0.01 | 0.03 | 0.04 |
| 1,4-dihydroxy-6-naphthoate biosynthesis I | 0.03 | 0.04 | 0.01 | 0.03 | 0.03 |
| mannan degradation | 0.23 | 0.13 | 0.15 | 0.13 | 0.03 |

1: MHO and Con subjects; 2: MUO subjects; MUO: metabolic unhealthy obese; MHO: metabolically healthy obese.

Table S6 KEGGs biomarkers in MHO and MUO subjects.

| pathway | 1: mean rel. freq. (%) | 1: std. dev. (%) | 2: mean rel. freq. (%) | 2: std. dev. (%) | p-values |
| --- | --- | --- | --- | --- | --- |
| superpathway of fucose and rhamnose degradation | 0.13 | 0.09 | 0.19 | 0.12 | 0.04 |
| photorespiration | 0.02 | 0.04 | 0.00 | 0.00 | 0.01 |
| sucrose degradation III (sucrose invertase) | 0.39 | 0.17 | 0.52 | 0.19 | 0.01 |

1: MHO subjects; 2: MUO subjects; MUO: metabolic unhealthy obese; MHO: metabolically healthy obese; LBP: lipopolysaccharide-binding protein.

Table S7 KEGGs biomarkers in MHO and Con subjects.

| pathway | 1: mean rel. freq. (%) | 1: std. dev. (%) | 2: mean rel. freq. (%) | 2: std. dev. (%) | p-values |
| --- | --- | --- | --- | --- | --- |
| arginine, ornithine and proline interconversion | 0.02 | 0.02 | 0.03 | 0.02 | 0.03 |
| dTDP-L-rhamnose biosynthesis I | 0.68 | 0.16 | 0.78 | 0.13 | 0.01 |
| adenosine nucleotides degradation IV | 0.00 | 0.00 | 0.00 | 0.00 | 0.02 |
| superpathway of adenosine nucleotides de novo biosynthesis II | 0.75 | 0.10 | 0.80 | 0.08 | 0.03 |
| chondroitin sulfate degradation I (bacterial) | 0.07 | 0.07 | 0.15 | 0.12 | 0.01 |
| adenosine deoxyribonucleotides de novo biosynthesis II | 0.66 | 0.16 | 0.76 | 0.15 | 0.03 |
| guanosine deoxyribonucleotides de novo biosynthesis II | 0.66 | 0.16 | 0.76 | 0.15 | 0.03 |
| superpathway of UDP-glucose-derived O-antigen building blocks biosynthesis | 0.10 | 0.07 | 0.15 | 0.07 | 0.03 |
| superpathway of pyridoxal 5'-phosphate biosynthesis and salvage | 0.14 | 0.08 | 0.22 | 0.10 | 0.01 |
| mycothiol biosynthesis | 0.00 | 0.00 | 0.00 | 0.00 | 0.03 |
| pyridoxal 5'-phosphate biosynthesis I | 0.11 | 0.07 | 0.17 | 0.09 | 0.01 |

1: MHO subjects; 2: Con subjects; MHO: metabolically healthy obese; Con: controls.
